# Phosphoproteome landscape of ARID1A and its implications in DNA damage response and breast cancer pathogenesis

**DOI:** 10.1007/s12672-025-03408-z

**Published:** 2026-01-22

**Authors:** Apoorva Pai Kalasa Anil Kumar, Leona Dcunha, Althaf Mahin, Prathik Basthikoppa Shivamurthy, Suhail Subair, Athira Perunelly Gopalakrishnan, Athira C. Rajeev, Rajesh Raju

**Affiliations:** Centre for Integrative Omics Data Science, Yenepoya (Deemed to be University), Mangalore, 575018 Karnataka India

**Keywords:** ARID1A, Phosphoproteomics, DNA damage response, Breast cancer

## Abstract

ARID1A, a subunit of the SWI/SNF chromatin-remodeling complex, plays a critical role in maintaining genomic stability and regulating estrogen receptor (ER) signaling, yet its phosphorylation dynamics in cancer remain underexplored. This study employed phosphoproteomic analysis to investigate ARID1A phosphorylation in breast cancer, identifying predominant phosphosites—S363, S1184, and S696—regulated by kinases such as MAPK14, CDK16, and MAPK9. Functional enrichment revealed ARID1A interactions with SWI/SNF components (e.g., PBRM1, SMARCC2, BRD9) and DNA damage response (DDR) proteins (e.g., TP53BP1, TOP2A, CHEK2, NBN), underscoring its dual role in chromatin remodeling and double-strand break repair. Notably, phosphorylation at S363 and S1184 was significantly upregulated in breast cancer, suggesting tumour-specific hyperphosphorylation that may disrupt ARID1A tumour-suppressive function and contribute to endocrine resistance. Dysregulation of these phosphorylation events correlated with enhanced MAPK signaling, cancer progression, and poor prognosis. These findings position ARID1A as a molecular hub linking chromatin dynamics to genome integrity, with implications for therapeutic resistance. Targeting ARID1A phosphorylation pathways, potentially via MAPK inhibitors or BRD4/BRD9 antagonists, could restore its suppressive activity and improve treatment outcomes in breast cancer. This study enhances our understanding of ARID1A regulatory mechanisms, highlighting its phosphorylation as a key driver of breast cancer biology. Future research should validate these kinase-substrate interactions and explore their transcriptional and chromatin-level impacts to develop precision therapies for ARID1A-dysregulated cancers.

**Supplementary Information:**

The online version contains supplementary material available at 10.1007/s12672-025-03408-z.

## Introduction

ARID1A (AT-rich interactive domain-containing protein 1 A), also known as BAF250a, SMARCF1, or p270, is a key member of the ARID protein family with a highly conserved DNA-binding domain [1]. As a core component of the BRG1-associated factor (BAF) complexes a subfamily of mammalian SWI/SNF chromatin remodelers. ARID1A, alongside its paralog ARID1B, plays a critical role in essential cellular processes [2]. These include transcriptional regulation, DNA replication, DNA damage repair (DDR), and the maintenance of genomic stability, cell proliferation, and apoptosis [2–4]. The N-terminal ARID domain of ARID1A preferentially binds AT-rich DNA sequences within the major groove, facilitating nucleosomal remodeling and gene transcription regulation, thus positioning ARID1A as a vital regulator of cellular homeostasis [5].

Located on chromosome 1p35.3, ARID1A is frequently mutated in various cancers, notably in breast cancer [6]. As a tumor suppressor, ARID1A inactivation is linked to tumor growth, therapy resistance, and poor prognosis [7]. Loss of ARID1A function disrupts chromatin remodeling, promoting cancer cell proliferation and resistance to treatments such as endocrine therapy and chemotherapy [6]. In ER + breast cancer, reduced ARID1A expression correlates with diminished treatment response, the emergence of resistant tumors, and shorter disease-free and overall survival, particularly in luminal A and HER2-enriched subtypes [8]. Furthermore, ARID1A loss in metastatic lesions is associated with resistance to therapies like trastuzumab and mTOR inhibitors, highlighting its role in driving resistance and tumor progression through increased tumor mutation burden [9].

The function of ARID1A is intricately regulated by post-translational modifications, with phosphorylation being a key modulator. Phosphorylation influences ARID1A interactions with other proteins, its chromatin-binding affinity, and its stability [10]. For instance, phosphorylated ARID1A interacts with β-TrCP, triggering ubiquitination and degradation, underscoring the importance of phosphorylation in controlling cellular ARID1A levels [10]. Dysregulated phosphorylation can activate pathways like PI3K/AKT, enhancing cell proliferation, endocrine resistance, and tumorigenesis in breast cancer [11]. Understanding the phosphorylation status of ARID1A offers potential for targeted therapies, such as de-phosphorylation strategies or enhancing ARID1A activity to improve treatment outcomes in breast cancer.

Given the dual role of ARID1A in DNA damage response (DDR) and endocrine resistance, we investigated its phosphoproteome landscape to uncover signatures relevant to breast cancer pathogenesis. By characterizing differentially regulated phosphopeptides—marked by hyper- or hypo-phosphorylation, we identified biologically significant phosphosites. These sites were selected based on their co-regulation with other phosphosites within interacting domains, shedding light on the contributions of ARID1A to DDR and breast cancer pathogenesis. This phosphoproteomic approach provides a foundation for understanding molecular regulation of ARID1A and its therapeutic implications in breast cancer.

## Materials and methods

### Data collection and compilation of phosphoproteomic datasets of ARID1A

To thoroughly explore the phosphorylation profile of ARID1A, we performed an in-depth literature review using PubMed, employing the search terms “phosphoproteomics” OR “phosphoproteome.” We applied strict filters to exclude plant-related studies and review articles, focusing exclusively on high-quality, mass spectrometry-based phosphoproteomic datasets derived from human cell lines to ensure relevance to human biology. Through careful manual curation, we selected global phosphoproteome datasets reporting Class-1 phosphosites, defined as phosphorylation sites with a localization probability of ≥ 75% and an Ambiguity Score (A-score) > 13, indicating high confidence in their identification and precise localization.

The curation process targeted datasets containing ARID1A phosphopeptides, generated via enzymatic digestion with LysC and/or trypsin, commonly used proteases in phosphoproteomic studies. To systematically analyze these datasets, we categorized them into two groups based on experimental design: (1) profiling datasets, which captured all detected phosphopeptides under test and control conditions as independent profiles, providing a comprehensive view of phosphorylation events, and (2) differential datasets, which quantitatively compared phosphosite abundance between test and control conditions to identify significantly altered phosphopeptides. This classification allowed for a detailed examination of ARID1A phosphorylation dynamics across diverse biological contexts.

For differential datasets, we analyzed Class-1 phosphosites to detect significant regulatory changes, defining upregulation as a fold change ≥ 1.3 and downregulation as a fold change ≤ 0.76, with statistical significance assessed using study-specific thresholds (*p* < 0.05). These criteria ensured the identification of biologically meaningful phosphorylation events.

To achieve accurate and consistent annotation of proteins and phosphosites across datasets, we developed a tailored, in-house mapping strategy. For protein annotation, we mapped each identified protein to its corresponding gene symbol using the HUGO Gene Nomenclature Committee (HGNC) database, leveraging the latest HGNC release to ensure standardized and current gene nomenclature. This step was essential for unifying protein identifiers across datasets with varying naming conventions, enabling seamless downstream analyses.

For phosphosite annotation, we aligned each phosphosite to its corresponding UniProt accession using the UniProt database release from May 13, 2023. We created a custom mapping tool to process phosphoproteomic datasets, extracting details such as residue type (serine, threonine, or tyrosine), position in the protein sequence, and sequence context. This tool matched phosphosites to the correct UniProt protein sequence, accounting for isoforms, and included validation steps to resolve discrepancies, ensuring high mapping precision. To support further analysis, we enriched datasets with standardized metadata tags describing experimental and biological conditions, following a structured annotation framework inspired by [12].

**Fig. 1:**
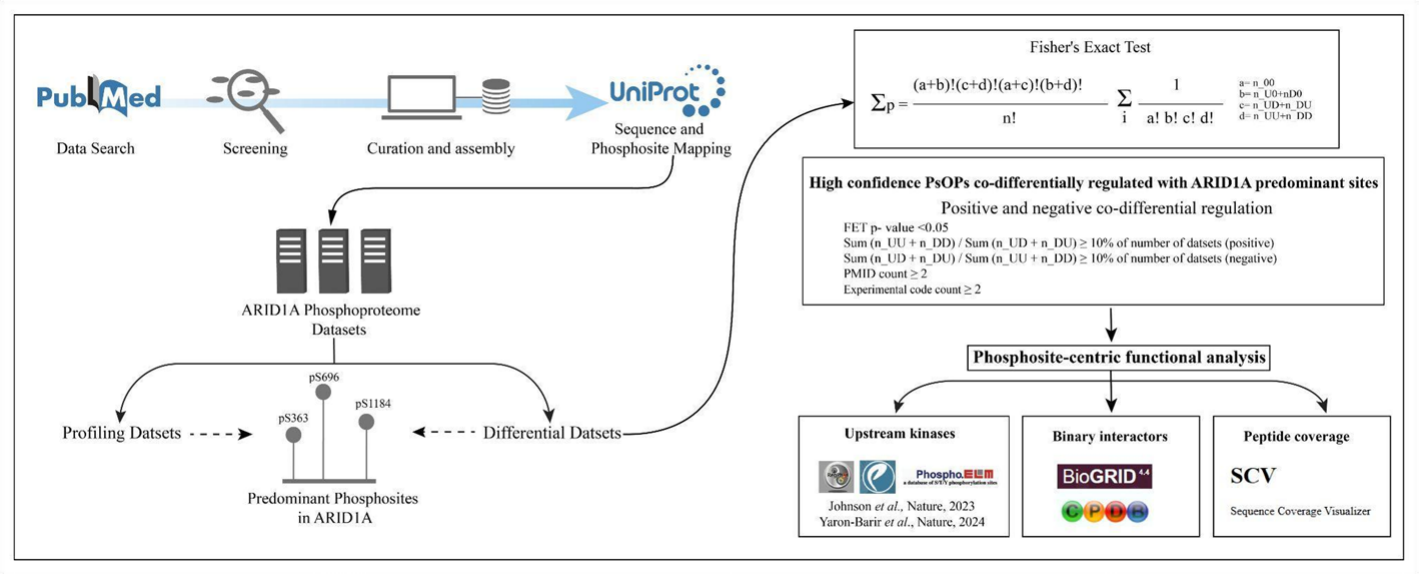
The workflow adopted in this study

This integrated approach—combining rigorous literature curation, clear dataset categorization, robust statistical thresholds, and precise mapping—provided a comprehensive framework for characterizing ARID1A phosphorylation, establishing a strong basis for elucidating its roles in human cellular functions. The workflow is depicted in Fig. 1.

### Identification of predominant phosphosites representing ARID1A in cellular phosphoproteome

From the compiled datasets, the number of qualitative profile datasets where the phosphosite is observed along with the quantitative datasets where the phosphosite is differentially regulated were computed and ranked. The sites identified to appear in more than 50% of the total datasets were chosen to represent the predominant phosphorylation status of ARID1A, hereafter mentioned as predominant phosphosites. The present criteria, however, prevented the inclusion of phosphosites that were not commonly identified as class-1 sites in these datasets but were found using techniques, such as phospho-antibodies or mutation-based approaches.

### Peptide coverage

To represent phosphorylation sites in the sequence and visualize ARID1A, Sequence Coverage Visualizer (SCV) [13] was utilized. Sequence coverage is one of the crucial metrics in bottom-up proteomics and it suggests the ratio of total observed protein sequence length to the overall protein sequence length. The modified peptide sequences identified from human phosphoproteomic cellular datasets were analyzed.

### Identification of phosphosites in other proteins co-differentially regulated with key ARID1A phosphosites

After identifying the predominant phosphosite, phosphosites of other proteins (PsOPs) that exhibited either positive or negative co-differential regulation with the predominant phosphosite of ARID1A were systematically categorized. Datasets were grouped and examined according to their quantitative profiles to find proteins with phosphosites (PsOPs) that co-phosphorylate positively or negatively with ARID1A predominant phosphosites (S696, S1184, S363). The following classifications were applied to each of these datasets. Phosphosites in other proteins (denoted as “o”) that were downregulated and upregulated with ARID1A (denoted as “a”) upregulation were designated as DoUa and UoUa. In contrast, those upregulated and downregulated with ARID1A downregulation were designated UoDa and DoDa. The PsOPs in UoUa and DoDa were considered positively co-regulated (in the same direction). In contrast, those in DoUa and UoDa were considered negatively co-differentially regulated (opposite direction) with the expression of ARID1A phosphosite. Furthermore, Fisher’s exact test (FET) was carried out. We sorted the data based on the criteria such as FET p-value < 0.05, then a ≥ 10% ratio of Ʃ(nUaUo + nDaDo)/Ʃ(nUaDo + nDaUo) for positively co-regulated PsOPs and the ratio of Ʃ(nUaDo + nDaUo)/Ʃ(nUaUo + nDaDo) for negatively co-regulated phosphosites; a minimum of 3 distinct experimental conditions (experimental code count) that support positive or negative regulation; and a minimum of 3 distinct articles (PubMed IDs) that support positive or negative regulation. The PsOPs filtered using the above-mentioned criteria were considered high-confidence proteins and used for subsequent analysis.

### Identification of putative upstream kinases, binary interactors and phosphatases of ARID1A among the PsOPs

To construct a comprehensive dataset of experimentally validated upstream kinases and their corresponding phosphosites for ARID1A, we integrated data from several databases, including PhosphoSitePlus (accessed May 22, 2023; [14]), Phospho.ELM 9.0 (accessed May 24, 2023; [15]) and RegPhos 2.0 (accessed May 24, 2023; [16]). To complement these findings, we employed in silico tools to predict ARID1A-specific upstream kinases, utilizing NetworKIN (predictions conducted January 4, 2023; [17]), AKID (predictions conducted May 24, 2023; [18]), and iKiP-DB [19] for kinase-substrate relationship analysis. Complete RefSeq protein sequences were processed via API support, as required by most prediction tools. Additionally, we included upstream kinases identified through synthetic peptide screening by Johnson et al. 2023 [20] targeting functional phosphosites reported by Ochoa et al. 2020 [21] with a 90th percentile cutoff.

For binary interaction data related to ARID1A, we aggregated information from multiple sources, including HPRD [22], BIND [23], BioGRID [24], ConsensusPathDB version 35 (accessed May 22, 2023; [25]), CORUM (accessed March 3, 2023; [26]) and RegPhos 2.0 (accessed May 24, 2023; [16]). Furthermore, to identify additional kinases and phosphatases within co-phospho-regulated networks, we utilized resources such as KinBase (https://www.hsls.pitt.edu/obrc/index.php? page=URL10548429580*)*, KinHub (http://www.kinhub.org/), and DEPOD (The Human DEPhOsphorylation Database [27]).

### Identification of phosphorylation motifs in kinases

Identification of phosphorylation motifs within kinases aid in the identification of phosphorylation sites, prediction of possible substrates, and comprehension of kinase specificity, which in turn aids in understanding signaling pathways and possible therapeutic targets. To this end, after identifying the upstream kinases of each of the predominant phosphosites of ARID1A from the FET data, a motif analysis was carried out using Phosphositeplus. The proteins which were positively co-phosphorylated with ARID1A predominant phosphosites in Her2 + and Her2- conditions were grouped and analyzed for motifs. The sequence pattern of ± 7 amino acids from the central position were submitted to the sequence logo tool in Phosphositeplus and sequences with similar phosphorylation motifs were identified.

### Tools utilized for data visualization

A variety of software and tools were employed to visualize the data collected in this research. Lollipop graphs were generated using track Viewer, a program available in R/Bioconductor (10.18129/B9.bioc.trackViewer). For enrichment analysis, the g: profiler tool [28] was utilized. The distribution of phosphosites across various data sets was illustrated using Pandas and Python Matplotlib. Interaction maps were visualized with Cytoscape (version 3.10.3) [29] and Pathvisio3 [30] while dendrograms and bar graphs representing gene ontology-enriched concepts were created using RAW Graph 2.0 (10.1145/3125571.3125585).

## Result and discussion

### ARID1A phosphoproteome data statistics

Mass spectrometry-based phosphoproteomics datasets were thoroughly analyzed to reduce variability in identifying Class-1 phosphosites of ARID1A across various biological and experimental conditions. By screening over 3,825 publicly available studies, we sourced 382 differential and 961 profiling datasets that reported the expression of ARID1A class 1 phosphosites in various human cell lines. The data of class-1 phosphosites in profile and differential datasets are provided in (Supplementary data 1 and 2). From this data, a few of the phosphosites which were observed in different experimental datasets were ranked based on their detection frequency. Notably, approximately 60% (577) of the qualitative profile datasets were derived from breast cancer cell lines, prompting a focused analysis on breast cancer alongside the broader global phosphoproteome perspective.

### S696, S1184 and S363 identified as predominant phosphosites of ARID1A

**Fig. 2:**
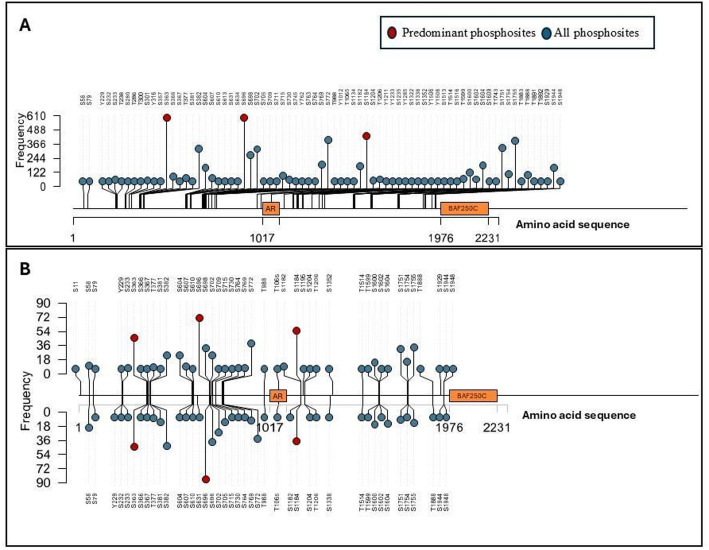
-Lollipop plot visualization of ARID1A phosphosite frequency. **A** Class-1 phosphosites found in ARID1A from quantitative profiling datasets taken globally. **B** The Y-axis represents the frequency of phosphosites found throughout the differential datasets, while the X-axis shows the phosphosites mapped onto the ARID1A amino acid sequence together with its structural domains. Positive values on the Y-axis indicate upregulation of phosphosite abundance, while negative values indicate downregulation

Through an analysis of 961 cellular qualitative profiles and 382 quantitative differential profile datasets, ARID1A phosphosites were scored based on detection frequency and differential regulation. This led to the identification of key phosphorylation sites on ARID1A (S696) (detected in 160 experimentally different conditions), (S1184) (detected in 160 experimentally different conditions), (S363) (detected in 94 experimentally different conditions) (Fig. 2). These phosphosites showed differential expression in phosphoproteomic studies related to lung, breast and pancreatic cancers, neuroblastoma and melanomas. The identified phosphosites were also documented in PhosphoSitePlus, reinforcing the viability of our findings. While specific functional annotation/role of the ARID1A S696 phosphosite remain unreported, it is notable that Pre-mRNA Processing Factor 4 kinase (PRP4K) is identified as a regulatory protein which can directly/indirectly affect the for ARID1A S696 phosphorylation status ([31], Supplementary Table 2). Serine/threonine kinase, PRP4K, is a key component of the U4/U6-U5 tri-snRNP complex, essential for pre-mRNA splicing and spliceosome assembly. Research by Islam et al. and 24,003,220 underscores the significance of PRP4 in cancer, highlighting its role in promoting epithelial-mesenchymal transition (EMT) and inducing drug resistance during cancer development and progression [32]. A study by Corkery et al., establishes that PRP4K correlates positively with HER2 expression in breast cancer and is a predictive biomarker for taxane response, with reduced PRP4K levels linked to taxane resistance [33].

Similarly, the S363 phosphosite is reported to be directly/indirectly regulated in vitro by cyclin-dependent kinase 2 (CDK2) ([34], Supplementary Data 3). CDK2 is also a serine/threonine kinase known to play a critical role in cell cycle progression, particularly in the G1/S phase transition, by phosphorylating key substrates like the retinoblastoma protein (Rb). Caldon and Musgrove report the cyclin E/CDK2 axis in breast cancer, highlighting its role in proliferation and therapeutic resistance, especially in tumors with cyclin E amplification [35].

For the S1184 phosphosite, no specific upstream kinase or functional data were detailed in this analysis. Notably, these predominant phosphosites—S696, S1184, and S363—are not associated with any known domains of ARID1A, and phosphosite-specific functional information remains limited, warranting further investigation into their biological significance. Additionally, the analysis of breast cancer data showed that among the identified predominant sites, only S363 and S1184 were detected, with S363 exhibiting the highest detection frequency.

### Sequence coverage of phosphopeptides of ARID1A

**Fig. 3:**
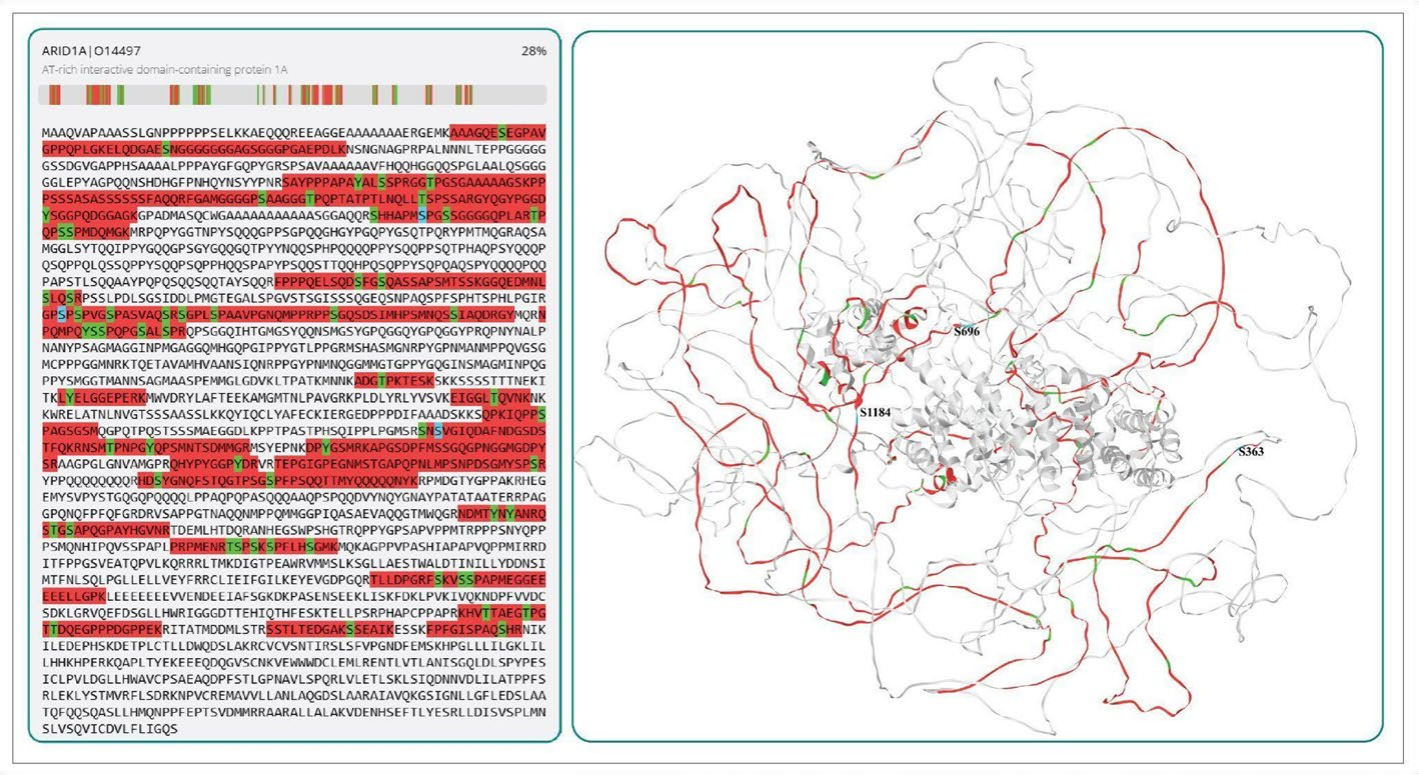
Sequence coverage of ARID1A and predominant phosphosite peptide detection from global phosphoproteomics data.The 3D-predicted ARID1A model represents peptides found in 7832 human cellular phosphoproteomic profiling datasets. Sequence coverage is shown in red, while Ser/Thr/Tyr phosphorylation is in blue and all the reported phosphosites in green

To assess the detection frequency of ARID1A peptides in high-throughput studies, we compared sequence coverage at both the proteome and phosphoproteome levels. The predominant phosphosites—S363, S696, and S1184—were found to be distributed across ARID1A, with S363 located in the N-terminal region and S696 and S1184 situated in the central region. This region is known to mediate interactions with other proteins, influence protein localization, and regulate functional activity. Using the SCV tool, we visualized the sequence coverage and highlighted these key phosphosites based on profiling data (Fig. 3). The peptides identified from this analysis achieved 28% coverage of ARID1A, with phosphorylation events clearly represented.

### Phosphosites in other proteins showing co-differential regulation with key phosphosites of ARID1A

To extract high confidence phosphosites in other proteins (PsOPs) that are co-differentially regulated with ARID1A predominant phosphosites, we paired ARID1A phosphosites with those in other proteins. This analysis relied on stringent criteria: a p-value < 0.05 calculated using Fisher’s exact test (FET), a ratio of ≥ 15% for either the number of positive co-phosphorylations to negative co-phosphorylations (n(UaUoDaDo/UaDoDaUo)) or the reverse (n(UaDoDaUo/UaUoDaDo)), evidence from at least three distinct experimental conditions, and support from a minimum of three independent studies confirming the co-phosphorylation.

**Fig. 4:**
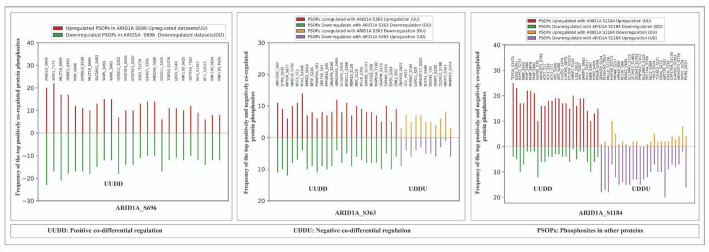
Top 25 high confidence proteins which positively and negatively co-phosphorylated with ARID1A (S1184) and ARID1A (S363). Co-regulation patterns are indicated by letter codes: U = Upregulated; D = Downregulated. For example, UUDD indicates Upregulated, UDDU indicates Downregulated

Our analysis revealed 50, 434, and 266 high-confidence proteins positively co-differentially regulated with ARID1A phosphosites S696, S1184, and S363, respectively (Supplementary Data 3–7). Additionally, 28 and 11 high-confidence proteins showed negative co-phosphorylation with S1184 and S696, respectively. However, S363 exhibited no negatively co-phosphorylated high-confidence proteins, as it did not meet the established cutoff criteria. The top 25 high confidence PsOPs co-differentially regulated with the most prevalent ARID1A phosphosites are presented with high confidence in Fig. 4, offering a clear visualization.

To elucidate the broader regulatory network surrounding ARID1A phosphorylation, we identified co-differentially regulated phosphosites in other proteins (PsOPs) associated with ARID1A predominant phosphosites: S696, S1184, and S363. Among the top PsOPs linked to ARID1A (S696) were WEE1-like protein kinase (WEE1) at T173 and protein AF-10 (MLLT10) at S689. For ARID1A (S1184), nibrin (NBN) at S343 and heat shock protein beta-1 (HSPB1) at S78 emerged as leading candidates, while ARID1A (S363) was associated with zinc finger protein 280 C (ZNF280C) at S80, regulatory-associated protein of mTOR (RPTOR) at S877, and maternal embryonic leucine zipper kinase (MELK) at S356 (Fig. 4). Notably, many of these proteins are established contributors to breast cancer pathogenesis, suggesting a functional interplay with ARID1A phosphorylation.

WEE1, a kinase pivotal to DNA damage response (DDR), regulates the G2–M checkpoint to ensure mitotic fidelity. It is overexpressed in multiple malignancies, including breast cancer, melanoma, leukemia, and brain tumors across pediatric and adult populations [36]. This overexpression correlates with tumor progression, implicating ARID1A (S696) in DDR pathways. Similarly, MLLT10, a transcriptional regulator, modulates higher-order chromatin structure and DDR through interactions with DNA repair and ATP-dependent chromatin remodeling factors [37]. Although the functional roles of WEE1 (T173) and MLLT10 (S689) remain uncharacterized, their co-regulation with ARID1A (S696) suggests potential contributions to chromatin dynamics in cancer. For ARID1A (S1184), NBN (S343), a core component of the MRN complex, is essential for DNA recombination, meiosis, telomere maintenance, and double-strand break (DSB) repair [38]. The S343 phosphosite undergoes phosphorylation and acetylation, contributing to enzyme activation, DNA repair, and apoptosis [39–42]. This association positions ARID1A (S1184) within DSB repair mechanisms. HSPB1 (S78), a stress-responsive protein, regulates cell survival, apoptosis, and cytoskeletal organization, serving as a prognostic biomarker in breast cancer [43]. HSPB1 knockdown impairs cell migration and enhances apoptosis, with S78 identified as an activation site [44, 45]. Its co-regulation with ARID1A (S1184) suggests involvement in stress response and survival pathways in cancer cells. Regarding ARID1A (S363), ZNF280C (S80), a zinc finger protein, modulates transcription and responds to DNA damage by regulating chromatin factors [46]. Although S80 lacks functional annotation in PhosphoSitePlus, TANK-binding kinase 1 (TBK1) is its reported regulator, with elevated TBK1 expression linked to tamoxifen resistance in breast cancer [47]. This connection implies ARID1A (S363) may influence endocrine resistance via ZNF280C. RPTOR (S877), a subunit of the mTORC1 complex, drives cell growth, proliferation, and survival, with overexpression associated with aggressive tumor behavior and poor prognosis in breast cancer [48]. The S877 phosphosite regulates cell cycle progression [49], suggesting ARID1A (S363) modulates mTOR signaling. MELK (S356), a serine/threonine kinase, supports cell cycle regulation, stem cell maintenance, and cancer cell survival. Frequently overexpressed in cancers, MELK correlates with poor prognosis, immune infiltration, and enhanced response to neoadjuvant chemotherapy (NAC) in ER-positive breast cancer [50]. Its co-regulation with ARID1A (S363) points to roles in proliferation and therapy response.

These findings demonstrate that PsOPs co-differentially regulated with ARID1A phosphosites- S696, S1184, and S363 are intricately tied to cancer pathways, including DDR, cell cycle control, stress response, and therapy resistance. The prevalence of uncharacterized phosphosites (e.g., WEE1 T173, MLLT10 S689, ZNF280C S80) highlights a critical gap in functional knowledge, necessitating further investigation. Moreover, associations with breast cancer-specific processes—such as endocrine resistance (via TBK1/ZNF280C) and tumor aggression (via RPTOR/MELK)—suggest ARID1A phosphorylation acts as a regulatory hub. These insights underscore the therapeutic potential of targeting ARID1A and its co-regulated PsOPs, while emphasizing the need for detailed mechanistic studies to clarify their roles in breast cancer progression and treatment response.

### Upstream kinases and phosphatases predicted among the PsOPs of ARID1A

**Fig. 5:**
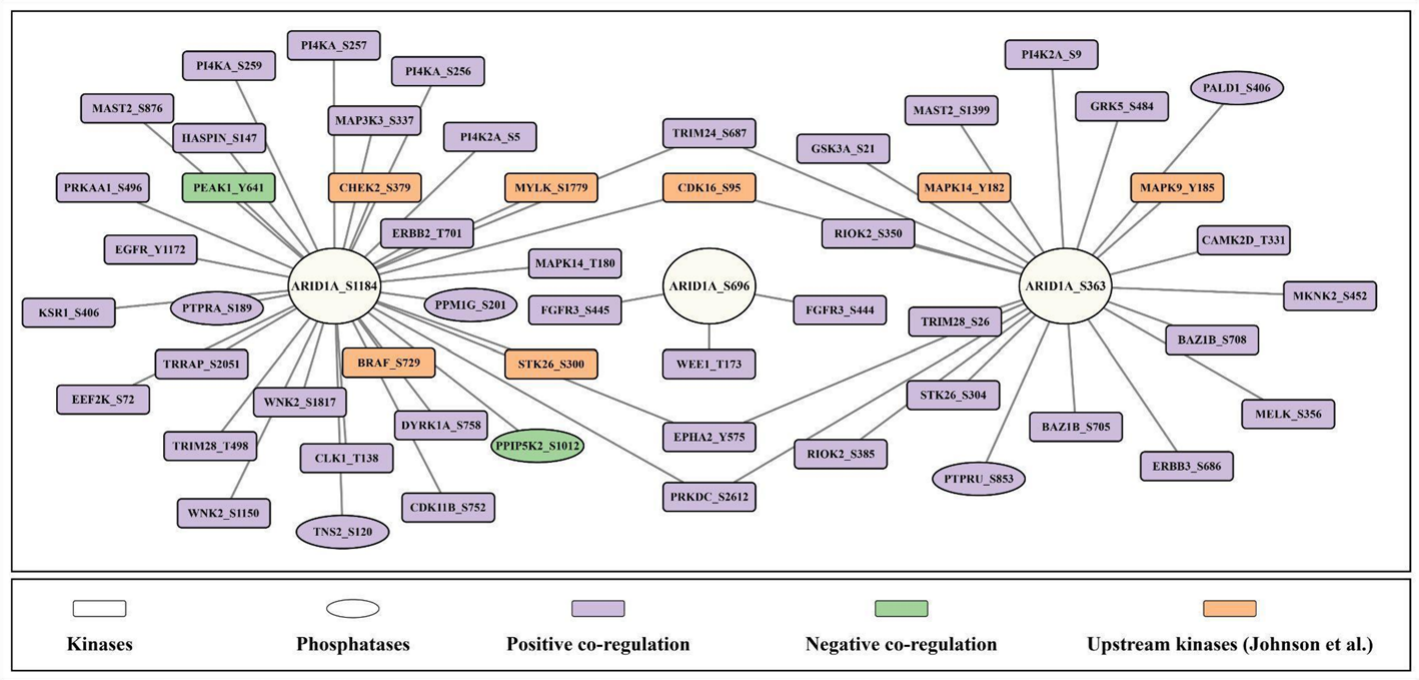
Network of selected kinases, phosphatases among the positively and negatively co-differentially regulated ARID1A phosphosites

Following the identification of co-differentially regulated phosphosites in other proteins (PsOPs) associated with ARID1A, we sought to predict upstream kinases regulating ARID1A phosphosites to uncover potential signaling pathways. Using the prediction method described by Johnson et al., we identified serine/threonine-protein kinase Chk2 (CHEK2), serine/threonine-protein kinase B-Raf (BRAF), myosin light chain kinase (MYLK), and serine/threonine-protein kinase 26 (STK26) as upstream kinases for ARID1A (S1184). The phosphosites CHEK2 (S379), BRAF (S729), MYLK (S1779), and STK26 (S300) were found to be positively co-phosphorylated with ARID1A (S1184). For ARID1A (S363), three upstream kinases were predicted: mitogen-activated protein kinase 14 (MAPK14), cyclin-dependent kinase 16 (CDK16), and mitogen-activated protein kinase 9 (MAPK9), with their respective phosphosites MAPK14 (Y182), CDK16 (S363), and MAPK9 (S95) showing co-differential regulation. No upstream kinases were identified for ARID1A (S696) within the available datasets. These relationships are depicted in Fig. 5.

Among the identified putative upstream kinases, CHEK2, BRAF, MYLK, MAPK14 and MAPK9 are reported in various breast cancer studies. CHEK2, a key regulator of DNA repair, cell cycle progression, and apoptosis, responds to DNA damage signals [51]. Its activation site, S379, suggests a role in coordinating DDR, potentially linking ARID1A (S1184) to genome stability pathways. Weischer et al., 2008 establishes that CHEK2 1100delC mutation increases breast cancer risk 2- to 3-fold, particularly in ER-positive subtypes, with a 37% lifetime risk by age 70 using meta-analysis 18,172,190. Similarly, several studies have found an association between CHEK2 variants and ER-positive breast cancer [52, 53].

BRAF, a member of the Raf kinase family, drives ERK and MAPK signaling, with mutations such as V600E causing constitutive activation and promoting cancer cell proliferation [54]. The S729 phosphosite, an activation site, implies that ARID1A (S1184) may intersect with oncogenic MAPK signaling. Although the studies documenting the role of BRAF in breast cancer is limited, few recent studies underline the potential of BRAF as an emerging target for cancers including breast cancer [55–57]. MYLK, implicated in tumor progression via epidermal growth factor receptor signaling in osteosarcoma [58], has an uncharacterized S1779 phosphosite, leaving its specific role in ARID1A regulation unclear. STK26 modulates gene expression by phosphorylating transcription-regulating proteins [59], suggesting a transcriptional regulatory link with ARID1A (S1184). These kinases collectively point to ARID1A (S1184) as a node in DNA repair, proliferation, and transcriptional control pathways. Literature studies reporting the correlation of STK26 and MYLK with breast cancer are limited to few studies like [60, 61] thereby demanding more experimental studies exploring the same.

MAPK14, a p38 MAPK family member, mediates cellular responses to cytokines and stress, influencing differentiation, inflammation, cancer progression, DDR, and oxidative stress [62]. It reportedly plays a dual role in several cancers, acting as both promoter and suppressor of tumor growth. Few studies have demonstrated that MAPK14 promotes the incidence and progression of breast cancer [63, 64]. Moreover, MAPK14 has also been shown to act as a suppressor in some malignancies, such as liver, colon, and lung cancer [65–67].

CDK16, associated with tumor growth [68], and MAPK9, a prognostic marker in glioma linked to tumor progression [69], further implicate ARID1A (S363) in cell cycle regulation and cancer development. The co-regulation of these kinases with ARID1A (S363) suggests involvement in stress response and proliferative pathways, potentially relevant to breast cancer pathogenesis. Apart from studies like [70] which reports the role of CDK16 in promoting the progression and metastasis of triple-negative breast cancer by phosphorylating PRC1, not many experimental studies are available stating the correlation between CDK16 and breast cancer. We also identified phosphatases co-differentially regulated with ARID1A phosphosites. For ARID1A (S1184), receptor-type tyrosine-protein phosphatase alpha (PTPRA) (S189), protein phosphatase 1G (PPM1G) (S201), and tensin-2 (TNS2) (S120) were positively co-regulated, while inositol hexakisphosphate and diphosphoinositol-pentakisphosphate kinase 2 (PPIP5K2) (S1012), a bifunctional protein, was negatively co-regulated. For ARID1A (S363), paladin (PALD1) (S406) and receptor-type tyrosine-protein phosphatase U (PTPRU) (S853) were positively co-regulated. PTPRA regulates GDNF-dependent RET signaling and inhibits oncogenic RET mutants (e.g., MEN2A) [71], suggesting a tumor-suppressive role. PPM1G dephosphorylates 4E-BP1, influencing chromatin structure, RNA splicing, and p53-dependent DDR [72]. TNS2, a tumor suppressor, supports cell adhesion and migration [73]. Conversely, PPIP5K2 activates AKT/mTOR signaling, promoting DNA repair, cell division, and metastasis [74], with its negative regulation indicating an opposing effect on ARID1A (S1184). PALD1 regulates cell adhesion and motility [75], while PTPRU supports adhesion, migration, invasion, and survival [76]. These phosphatases highlight a balance of tumor-suppressive and oncogenic signals modulating ARID1A activity.

The predicted upstream kinases and co-regulated phosphatases reveal a complex regulatory landscape for ARID1A phosphorylation. For S1184, the involvement of CHEK2 and BRAF suggests integration into DDR and MAPK-driven proliferation pathways, respectively, with MYLK and STK26 adding potential links to tumor progression and transcriptional regulation. For S363, MAPK14, CDK16, and MAPK9 points to roles in stress response, cell cycle control, and tumor growth, aligning with known functions of ARID1A in cancer. The absence of upstream kinases for S696 underscores a knowledge gap requiring further exploration. The phosphatases, particularly PTPRA, PPM1G, and TNS2, suggest tumor-suppressive counter regulation, while the negative correlation of PPIP5K2 with S1184 hints at oncogenic antagonism.

These observations position ARID1A phosphorylation is likely relevant in cancer-related processes, including DNA repair, cell proliferation, and therapy resistance, with relevance to breast cancer. The functional ambiguity of several phosphosites (e.g., MYLK S1779, STK26 S300) necessitates further characterization to clarify their roles. Moreover, the interplay between kinases and phosphatases suggests a dynamic regulatory network that could be exploited therapeutically, such as targeting CHEK2 or BRAF to modulate ARID1A activity in DNA damage or proliferative contexts. Future studies should focus on validating these predictions experimentally and elucidating the mechanistic contributions of these phosphosites to ARID1A function and cancer progression.

### Binary interactors among the PsOPs of ARID1A

**Fig. 6:**
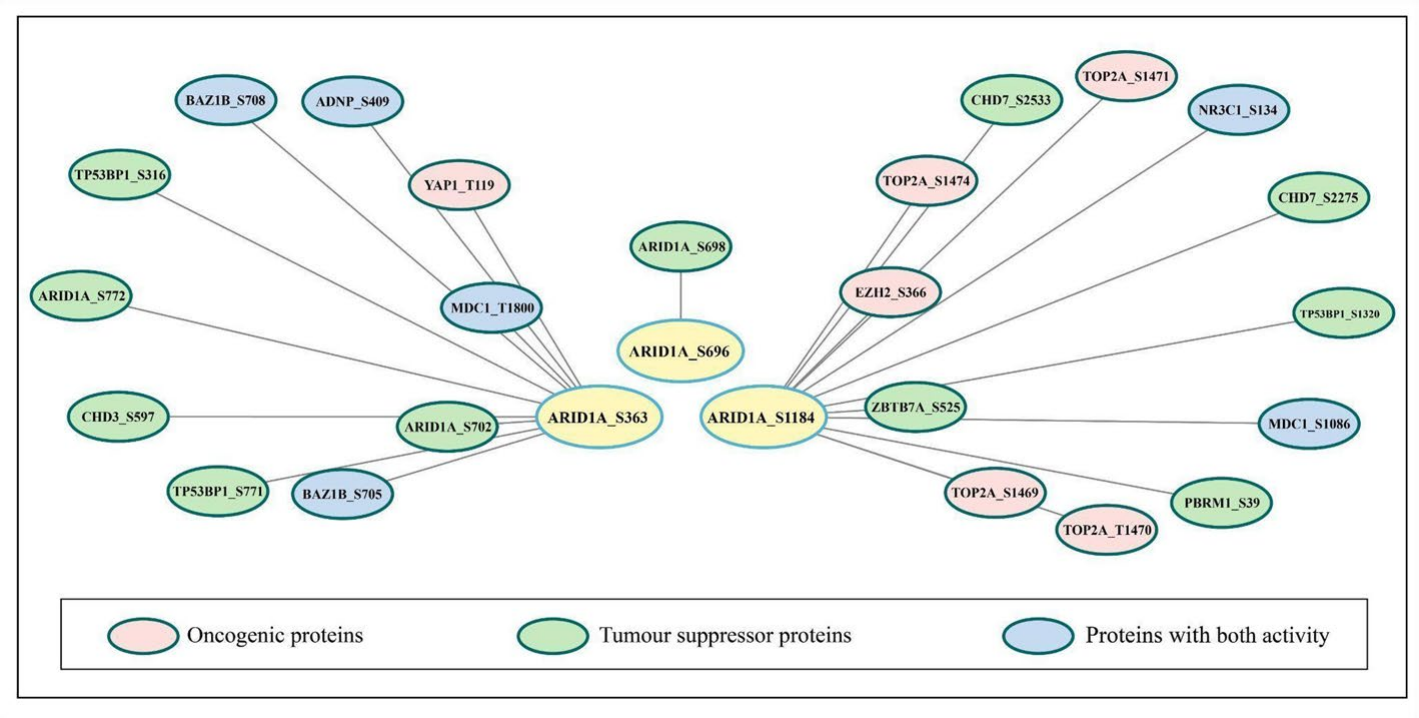
Selected binary interactors of ARID1A phosphosite-(S696) (S1184) (S363) which are reported as oncogenic, tumor suppressor and both

The co-regulation analysis was performed at the phosphosite level within these known protein–protein interaction pairs. To further explore the regulatory landscape of ARID1A phosphorylation, we predicted binary interactors for its three predominant phosphosites: S696, S1184, and S363. A total of 23 interactors were identified, with 1 associated with ARID1A (S696), 12 with ARID1A (S1184), and 10 with ARID1A (S363) (Supplementary Data 10). These interactors were classified into oncogenic, tumor suppressor, or dual-role categories and are illustrated in Fig. 6. Notably, ARID1A (S1184) exhibited the highest number of binary interactions, suggesting the relevance within ARID1A interaction network. Key interactors of ARID1A (S1184) included DNA topoisomerase 2-alpha (TOP2A) at phosphosites S1474, S1470, S1471, and S1469, TP53-binding protein 1 (TP53BP1) at S1320, and protein polybromo-1 (PBRM1) at S39. Each of these proteins is intricately linked to the DNA damage response (DDR). TOP2A, essential for DNA replication and repair, resolves DNA entanglements, with its phosphorylation sites implicated in regulating enzymatic activity during DDR [77]. TP53BP1, a critical DDR mediator, facilitates DNA double-strand break repair and checkpoint activation, with S1320 potentially modulating its repair functions [78]. PBRM1, a subunit of the SWI/SNF chromatin-remodeling complex, contributes to genome stability, and its S39 phosphorylation may enhance DDR coordination [79]. The enrichment of DDR-related interactors with ARID1A (S1184) underscores its pivotal role in DNA repair pathways.

The nuclear enzyme topoisomerase II alpha (TOP2A) is a critical regulator of the cell cycle and DNA topology, facilitating DNA replication, chromosome segregation during anaphase, and DNA decoiling. It achieves this by forming a transient protein gate that allows an intact DNA duplex to pass through a cleaved DNA segment [80]. This function is vital for maintaining genomic fidelity, particularly in rapidly dividing cells such as those in breast tissue. Tumor protein p53 binding protein 1 (TP53BP1) complements this system by mediating the DNA damage response, specifically in repairing double-strand breaks (DSBs) through non-homologous end joining (NHEJ) [81]. This repair pathway is essential for genomic stability in breast epithelial cells exposed to genotoxic stress. Additionally, polybromo-1 (PBRM1), encoded by a chromatin-remodeling gene, governs DNA structure and accessibility, playing a pivotal role in DSB repair [82]. Mutations in PBRM1 impair DNA repair capacity, increasing genetic instability, and a hallmark of breast cancer progression. However, the functional role of its phosphorylation site at serine 39 (S39) remains unexplored, representing a potential regulatory mechanism relevant to breast cancer. As a subunit of the SWI/SNF chromatin-remodeling complex, ARID1A likely interacts with TOP2A, TP53BP1, and PBRM1 to regulate DNA repair and chromatin dynamics in breast cells. The phosphorylation of ARID1A at S1184 may serve as a molecular switch, modulating DNA damage responses and chromatin accessibility processes frequently dysregulated in breast cancer.

These observations have significant implications for breast cancer biology. TOP2A overexpression is a well-documented feature in aggressive breast cancers, such as triple-negative breast cancer (TNBC), where it drives uncontrolled proliferation and is a target for chemotherapeutic agents like anthracyclines [80]. Similarly, TP53BP1 dysfunction, often linked to BRCA1-deficient breast tumors, shifts DSB repair toward error-prone pathways, fostering genomic instability and tumor progression [81]. PBRM1 mutations, while less studied in breast cancer compared to renal cell carcinoma, may contribute to chromatin remodeling defects that exacerbate DNA repair deficiencies, particularly in hormone receptor-negative subtypes [82]. The uncharacterized S39 phosphosite on PBRM1 could represent a novel regulatory element influencing repair efficiency in breast cancer cells under oxidative or replicative stress. Furthermore, ARID1A mutations are increasingly implicated in breast cancer, where they disrupt SWI/SNF-mediated gene regulation and DNA repair, promoting oncogenesis.

These findings suggest a tightly knit molecular network where dysregulation of TOP2A, TP53BP1, PBRM1, or ARID1A could synergistically indulge in breast cancer pathogenesis. For instance, elevated TOP2A activity combined with impaired TP53BP1 or PBRM1 function might amplify genomic instability in BRCA1/2-mutated breast tumors, accelerating malignancy. Likewise, altered ARID1A phosphorylation at S1184 could disrupt its coordination with these proteins, tipping the balance toward tumorigenesis in breast epithelial cells. These interactions highlight potential therapeutic opportunities, such as targeting TOP2A with inhibitors or exploiting synthetic lethality in PBRM1- or ARID1A-deficient breast cancers.

### Chromatin remodeling and double strand break repair-related proteins among the co-differentially regulated PsOPs

Among the co-differentially regulated proteins of ARID1A, numerous proteins reported to be associated with chromatin remodeling and double strand break repair were recognized. As an established regulator of chromatin remodeling and DNA damage response (DDR) pathways [83–85], the presence of these identified proteins among the co-differentially regulated proteins strengthens our results. Key interactors, including PBRM1 (S39), BCL7A, BRD7, DPF2, ARID2, PHF10, TP53BP1 (S1320), TOP2A (T1470, T1471, S1469, S1474), CHEK2 (S379), and NBN (S343, S397, S615), are critical for chromatin remodeling and DSB repair. PBRM1 at serine 39 (S39), a key subunit of the polybromo-associated BAF (pBAF) complex within the SWI/SNF chromatin-remodeling machinery, exhibited positive co-differential regulation with ARID1A (S1184). The SWI/SNF complex, encompassing subcomplexes such as canonical BAF (cBAF), non-canonical BAF (ncBAF), and pBAF, includes additional components like BCL7A, BRD7, DPF2, SMARCE1, ARID2, PHF10, SMARCA2, SMARCB1, SS18L1, SMARCD1, SMARCC1, and ACTL6A. Our data revealed a dense network of interactions among these SWI/SNF proteins.

Phosphorylation at ARID1A phosphosites S363, S696, and S1184 may induce precise alterations in the normal biochemical functions of the BAF complex by controlling its structural integrity, chromatin remodeling ability, and genomic targeting. Phosphorylation of S363 near the N‑terminus could modulate interaction of ARID1A with core BAF subunits like BRG1, potentially affecting complex assembly and stability, a mechanism consistent with ARID1A known role in recruiting and scaffolding such subunits. Phosphorylation at S696 may impact recruitment to specific genomic loci or alter nucleosome affinity, thereby influencing the complex’s capacity to remodel chromatin and maintain open enhancer landscapes—functions supported by the established role of ARID1A at enhancers In contrast, phosphorylation at the C‑terminal S1184 site may regulate the complex’s participation in DNA damage response pathways. ARID1A is known to be recruited to double-strand break sites via ATR and to facilitate chromatin relaxation during repair Furthermore, co‑differential regulation of PBRM1 at S39, a protein that tethers the PBAF complex to genomic loci, alongside ARID1A S1184 suggests a coordinated control mechanism linking phosphorylation events to broader SWI/SNF complex functionality[86].

Our analysis also identified several DSB repair proteins co-regulated with ARID1A (S1184), including TP53BP1 (S1320), TOP2A (T1470, T1471, S1469, S1474), CHEK2 (S379), NBN (S343, S397, S615), and LIG1 (S913, S911). These proteins were upregulated in concert with ARID1A.For instance, TOP2A, a nuclear enzyme that resolves DNA topological stress during replication and chromosome segregation, is modulated by phosphorylation at multiple sites [87]. Its dysregulation is a hallmark of aggressive cancer, including breast cancer, where it is both a prognostic marker and a therapeutic target. Similarly, CHEK2, a tumor suppressor kinase phosphorylated at S379 by ATM/ATR, activates DDR pathways and is implicated in breast cancer susceptibility when mutated [88]. NBN, a component of the MRN complex, facilitates DSB detection and repair, with its phosphorylation regulating DDR efficiency; mutations in NBN are linked to cancer predisposition [89]. LIG1, essential for NHEJ and replication, completes DNA repair through ligation, and its dysregulation contributes to genomic instability [90].

In the context of breast cancer, these findings carry significant implications. ARID1A mutations are prevalent in breast cancers, where they impair SWI/SNF-mediated gene regulation and DNA repair, fostering oncogenesis. The co-regulation of ARID1A (S1184) with TOP2A, overexpressed in triple-negative breast cancer (TNBC), suggests a synergistic role in driving proliferation and chemotherapy resistance [87]. Likewise, TP53BP1 and CHEK2 dysregulation in BRCA1/2-mutated breast tumors shifts repair toward error-prone NHEJ, amplifying genomic instability [81, 86]. Although PBRM1 mutations are less characterized in breast cancer, their role in chromatin remodeling and DSB repair, potentially modulated by S39 phosphorylation, could exacerbate DNA repair defects in aggressive subtypes like TNBC. The uncharacterized S39 site on PBRM1 may represent a novel regulatory switch influencing repair fidelity in breast cancer cells under stress.

In summary, our analysis identified several co-regulated proteins, including PBRM1 (S39), TP53BP1 (S1320), TOP2A (T1470, T1471, S1469, S1474), CHEK2 (S379), and NBN (S343, S397, S615), alongside ARID1A (S1184), that are critical for chromatin remodeling and DSB repair. These interactions, particularly within the SWI/SNF complex and DDR pathways, underscore a complex phosphorylation network driving genomic instability in breast cancer. The uncharacterized PBRM1 S39 site may represent a novel regulatory mechanism, warranting further investigation into its role in DNA repair fidelity under genotoxic stress.

### Co-differentially regulated PsOPs of ARID1A (S363) imply role in breast cancer pathogenesis

As mentioned in Sect. 3.1, the prevalence of breast cancer datasets among the global profile dataset prompted us to dive deeper into the probable role of ARID1A phosphorylation in breast cancer -related regulatory networks. Among its phosphorylation sites, ARID1A at serine 363 (pS363) exhibited significant correlation with breast cancer-related genes, MAPK14, ERBB3, ATF2. MAPK14 (p38 MAP kinase), phosphorylated at tyrosine 182 (Y182), emerges as a potential upstream kinase for ARID1A (pS363), showing a positive correlation in our data. MAPK14 is a therapeutic target in breast cancer, where its high expression drives tumor growth, proliferation, migration, and invasion, correlating with poor prognosis [91]. Phosphorylation at Y182 activates MAPK14 [92], which might affect the ARID1A-mediated chromatin remodeling and tumor suppression. This regulatory relationship suggests that MAPK14 may modulate ARID1A function, influencing breast cancer cell behavior. Similarly, ERBB3, a member of the epidermal growth factor receptor (EGFR) family phosphorylated at serine 686 (pS686), is frequently overexpressed in breast cancer. ERBB3 dimerizes with ERBB2 (HER2) to activate downstream signaling, promoting proliferation, survival, and metastasis while contributing to tamoxifen resistance in ER + subtypes [93]. Its positive correlation with ARID1A (pS363) hints at a crosstalk between ERBB3 signaling and ARID1A-mediated chromatin dynamics. Activating Transcription Factor 2 (ATF2), another interactor, shows a positive correlation with ARID1A (pS363). Recognized for its tumor-suppressive potential, elevated ATF2 levels may enhance sensitivity to endocrine therapies like tamoxifen in ER + breast cancer [94]. This interaction suggests that ARID1A (pS363) could influence treatment outcomes through ATF2 regulation. EPHA2, a receptor tyrosine kinase phosphorylated at tyrosine 575 (Y575), is overexpressed in ER + breast cancer and associated with aggressive behavior, including enhanced invasion, metastasis, and poor prognosis [95]. Its co-regulation with ARID1A (pS363) further underscores its role in breast cancer progression, potentially via shared signaling networks. These phosphorylation interactions converge on the MAPK signaling pathway, a central player in breast cancer. MAPK14 modulates estrogen receptor signaling and stress responses [96], while integration of ARID1A with MAPK pathways influences cancer development [97]. ERBB3 activates MAPK signaling, contributing to endocrine resistance [98], and ATF2, regulated by MAPK, affects tumor growth and therapy response [94]. EPHA2, also tied to MAPK signaling, drives cancer cell motility and invasion [99]. The co-regulation of ARID1A (pS363) with these proteins suggests that its phosphorylation may amplify MAPK-driven oncogenic processes in breast cancer.

**Fig. 7:**
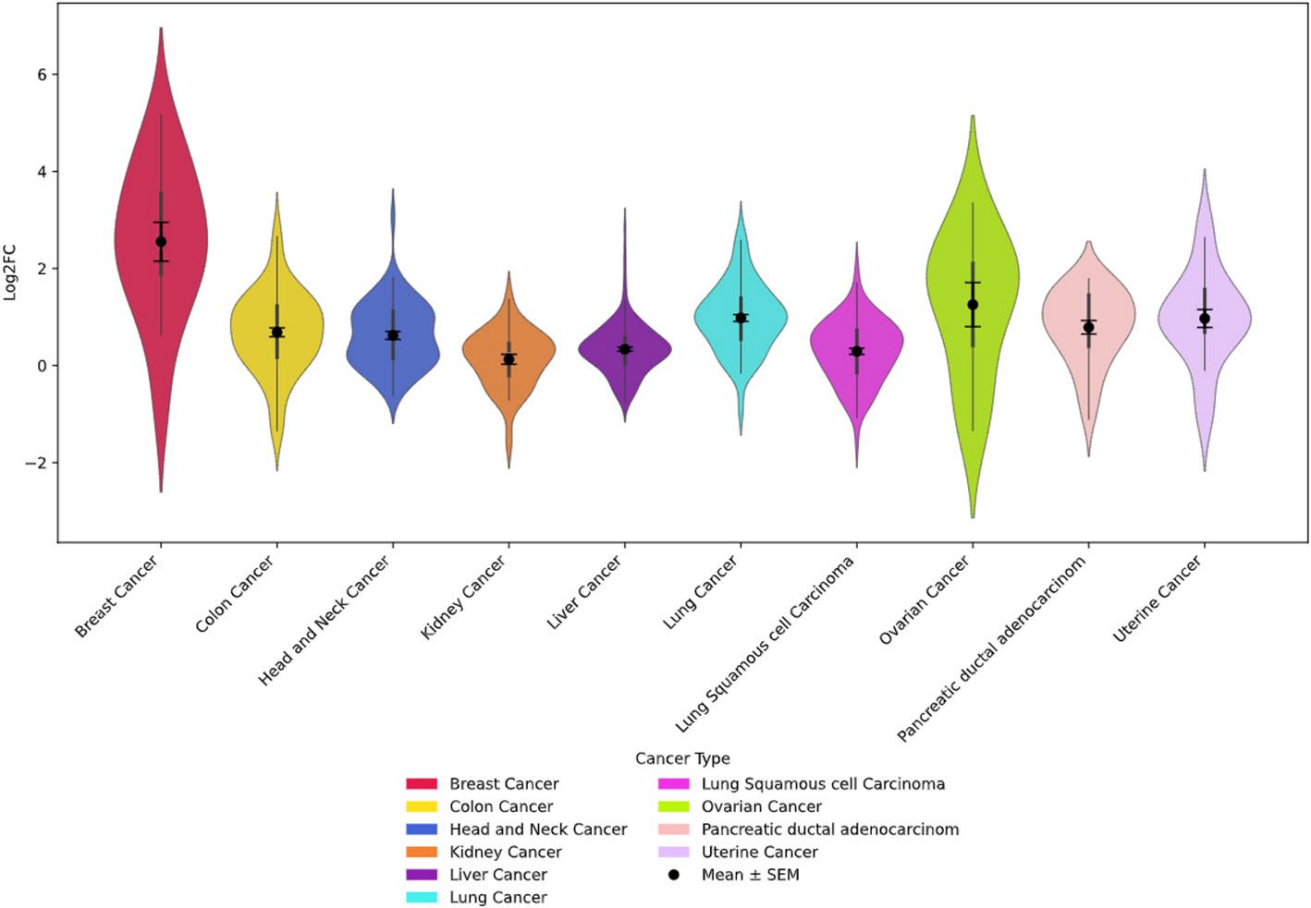
Violin plot illustrating the log2 fold change (Log2FC) distribution of co-differentially regulated proteins associated with ARID1A pS363 across various cancer types, including breast cancer, colon cancer, head and neck cancer, kidney cancer, liver cancer, lung squamous cell carcinoma, ovarian cancer, pancreatic ductal adenocarcinoma, and uterine cancer. Each violin represents the density of Log2FC values for a specific cancer type, with the mean ± standard error of the mean (SEM) indicated by black markers within each distribution. The plot highlights the variability in protein regulation across cancer types, with breast cancer showing the widest range of Log2FC values

To further investigate the correlation of the ARID1A predominant phosphosites with various cancers, c-ProSite tool was employed. ARID1A pS363 exhibits the largest fold change among its predominant phosphosites (S363, S1184, S696) in breast cancer, highlighting its tumor-specific significance (Fig. 7).

### Sequence motif analysis reveals similar motif-bearing proteins among the PsOPs

**Fig. 8:**
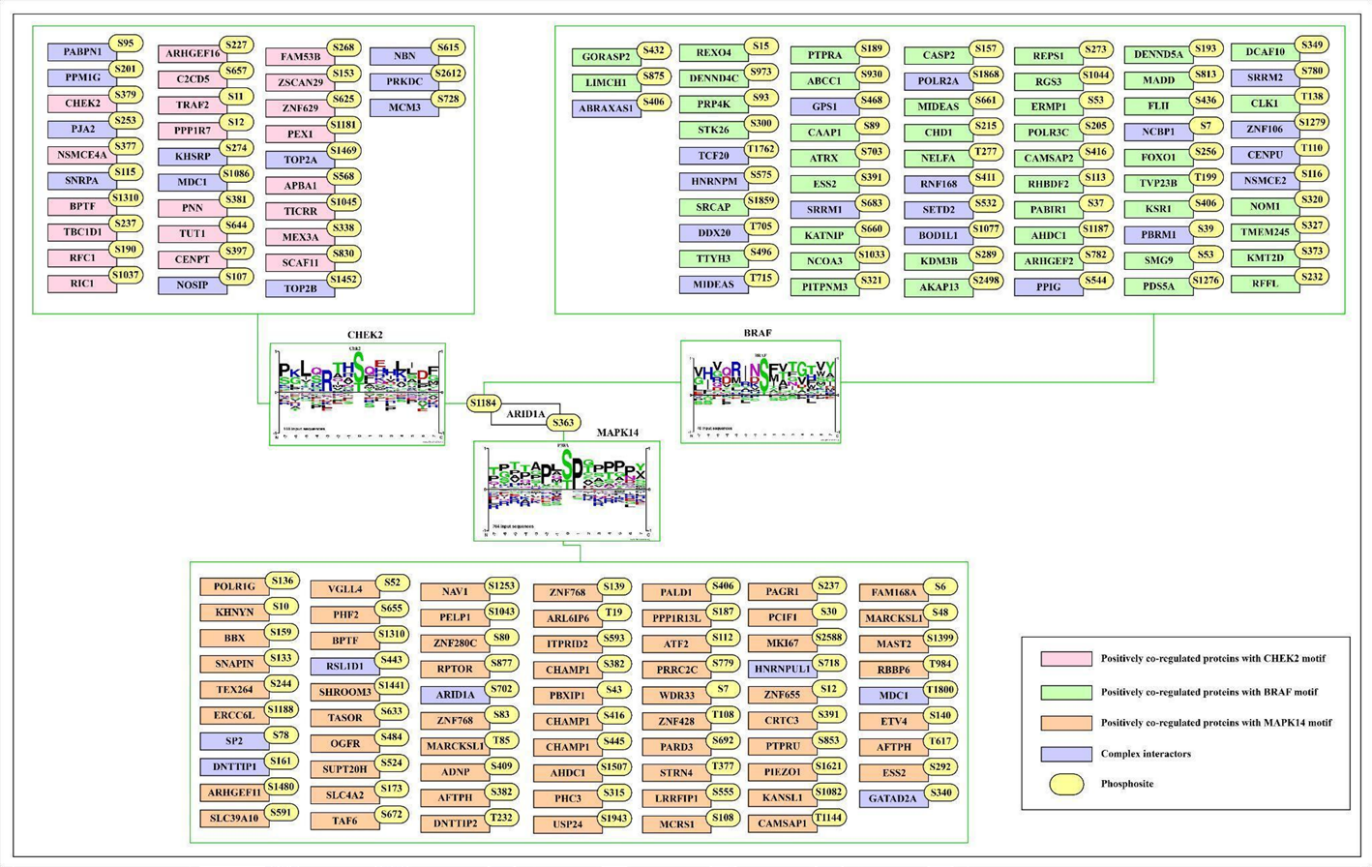
High correlated proteins which have similar motifs as that of upstream kinases

To investigate kinase-substrate associations (KSAs) for ARID1A phosphorylation, we utilized PhosphoSitePLUS as a reference dataset, which provided sequence logos for upstream kinases. Specifically, MAPK14 was identified as a candidate kinase for ARID1A at serine 363 (S363), while CHEK2 and BRAF were associated with serine 1184 (S1184). Proline-directed phosphorylation sites, such as those at S363 and S1184, are typically targeted by cyclin-dependent kinases (CDKs) or mitogen-activated protein kinases (MAPKs). We analyzed high-confidence proteins positively co-regulated within a ± 7 amino acid sequence pattern around these sites and compared them to the motifs of upstream kinases [20]. The resulting phosphomotif patterns suggest that MAPK14, CHEK2, and BRAF may phosphorylate ARID1A at S363 and S1184, alongside other co-regulated proteins, as depicted in Fig. 8.

In frequently mutated cancers like ovarian clear cell carcinoma, endometrial cancer, and gastric cancer, ARID1A loss is associated with tumor progression and poor prognosis [100]. In ER + luminal A breast cancer, ARID1A mutations or reduced expression disrupt estrogen receptor signaling and contribute to endocrine therapy resistance [101]. This loss impairs normal chromatin remodeling and gene regulation, promoting uncontrolled proliferation and survival. For instance, ARID1A depletion has been shown to enhance oncogenic PI3K/AKT signaling, further driving treatment resistance [100]. The complete crystal structure of ARID1A is not yet available and the partially solved structures are available in PDB [102]. The group based prediction system (https://gps.biocuckoo.cn/online_full.php) was used to predict the secondary structure along with the intrinsically disordered region. The predominant phosphosites- S363, S696, and S1184 were found to reside in the intrinsically disordered region outside defined domains. This observation was also supported by phosphositeplus [103-105].

Our analysis revealed expression-co-differentially regulated phosphosites in upstream kinases of ARID1A. For S1184, these included CHEK2 (S379), BRAF (S729), STK26, and MYLK, while for S363, MAPK14 (Y182), MAPK9, and CDK16 were prominent. Activation sites at CHEK2 (S379), BRAF (S729), and MAPK14 (Y182) indicate their functional significance. Given that proline-directed sites are characteristic of MAPKs and CDKs, we identified positively co-regulated proteins sharing similar motif patterns with these kinases, potentially implicating them in breast cancer pathogenesis. Experimental conditions further revealed that ARID1A (S1184) and (S696) phosphosites were associated with HER2+ and HER2- breast cancer contexts. Fisher’s Exact Test (FET) analysis highlighted MAPK14 (T180) as a positively co-regulated kinase with ARID1A (S1184), observed under conditions of JNK and p38 inhibition with anisomycin treatment in U2OS cells (Log2FC). This suggests that MAPK14 (T180) may act as an upstream kinase for ARID1A (S1184), with inhibitory regulation under specific stress conditions.

These findings illuminate a complex interplay between ARID1A phosphorylation, chromatin remodeling, and signaling pathways in breast cancer. The identification of MAPK14 as a potential kinase for both S363 and S1184 suggests a dual regulatory role, possibly modulating tumor-suppressive function of ARID1A in response to cellular stress or hormonal cues. In breast cancer, MAPK14-mediated activation of ARID1A (S363) could enhance proliferation and resistance to endocrine therapies like tamoxifen, consistent with its known role in estrogen receptor signaling dysregulation [80]. Similarly, CHEK2 and BRAF phosphorylation of S1184 may link DNA damage response and MAPK signaling to chromatin remodeling defects, amplifying genomic instability in endocrine-resistant tumors. The co-regulation of these phosphosites with HER2 + and HER2- contexts further implies subtype-specific roles, with S1184 potentially influencing HER2-driven pathways. Therapeutically, these observations suggest that targeting upstream kinases like MAPK14, CHEK2, or BRAF could restore ARID1A function and overcome endocrine resistance. For instance, p38 MAPK inhibitors might disrupt the MAPK14-ARID1A (S363) axis, sensitizing tumors to hormonal therapies. The inhibitory relationship between MAPK14 (T180) and ARID1A (S1184) under stress conditions also hints at context-dependent regulation, which could be exploited to modulate DNA repair or proliferation in resistant cells. Moreover, the predominance of these phosphosites in disordered regions underscores their flexibility as signaling hubs, potentially serving as biomarkers for stratifying patients with endocrine-resistant luminal A breast cancer.

In conclusion, our study highlights the phosphorylation dynamics of ARID1A at S363 and S1184, driven by kinases such as MAPK14, CHEK2, and BRAF, as critical regulators in breast cancer. These interactions bridge chromatin remodeling with oncogenic signaling, offering insights into tumor progression and treatment resistance. Future investigations should validate these KSAs using kinase assays or phosphomimetic mutants in breast cancer models, paving the way for targeted therapies to exploit this regulatory network.

### Mechanistic contributions of ARID1A phosphosites to tumorigenesis

To elucidate how ARID1A phosphorylation at S363, S696, and S1184 contributes to tumorigenesis in breast cancer, we investigated their mechanistic roles in disrupting SWI/SNF complex interactions and modulating alternative oncogenic pathways. These phosphosites, identified as predominant through phosphoproteomic analysis, reside in intrinsically disordered regions of ARID1A, which are critical for flexible protein interactions and signaling (Sect. 3.9). Below, we discuss their potential mechanisms based on their regional localization, co-regulated proteins, and upstream kinases.

The S363 phosphosite, located in the N-terminal region of ARID1A, is regulated by kinases such as MAPK14, CDK16, and MAPK9 (Sect. 3.5). This region is rich in serine, proline, and glutamine residues, facilitating interactions with SWI/SNF complex components (e.g., SMARCC2, BRD9) and other regulatory proteins. Phosphorylation at S363 may disrupt ARID1A’s recruitment to or stability within the SWI/SNF complex, impairing its chromatin remodeling function. For instance, hyperphosphorylation at S363, observed in breast cancer (Sect. 3.8), could weaken interactions with SMARCA4 or SMARCC1, reducing nucleosome repositioning and altering gene expression profiles critical for tumor suppression. This disruption may promote oncogenic transcription, enhancing cell proliferation and endocrine resistance, as seen with co-regulated proteins like RPTOR (S877) and MELK (S356), which drive mTOR signaling and cancer cell survival (Sect. 3.4). Additionally, S363’s co-regulation with MAPK14 (Y182) and ATF2 suggests integration with MAPK signaling, amplifying tumor growth and therapy resistance. (Sect. 3.8).

Phosphosites S696 and S1184, located in the central region of ARID1A, are near the ARID domain and glutamine-rich regions critical for protein-protein interactions and DNA-binding regulation. S1184 is regulated by kinases such as CHEK2 and BRAF, while no specific upstream kinases were identified for S696 (Sect. 3.5). These phosphosites show significant upregulation in breast cancer (Sect. 3.9), suggesting a role in oncogenic signaling. Phosphorylation at S696 and S1184 may enhance ARID1A interactions with DNA damage response (DDR) proteins, such as TP53BP1 (S1320), TOP2A (S1469, S1470, S1471, S1474), and NBN (S343, S397, S615), as identified in Sect. 3.7. This could aberrantly activate DDR pathways, promoting error-prone repair (e.g., via NHEJ) and genomic instability in breast cancer cells. For example, hyperphosphorylated S1184 may strengthen ARID1A coordination with PBRM1 (S39) within the SWI/SNF complex, altering chromatin accessibility and facilitating oncogenic gene expression. Alternatively, S696 and S1184 may modulate MAPK signaling through BRAF (S729) and MAPK14 (T180), enhancing proliferation and endocrine resistance, as seen with co-regulated ERBB3 (S686) in breast cancer (Sect. 3.8).

Beyond SWI/SNF disruption, ARID1A phosphorylation at these sites likely drives tumorigenesis through alternative pathways, including PI3K/AKT and MAPK signaling. We observed that ARID1A dysregulation may influence PI3K/AKT signaling (Sect. 1), which is supported by S363 co-regulation with RPTOR (S877), a component of mTORC1 (Sect. 3.4). This suggests that S363 phosphorylation may activate mTOR signaling, promoting cell growth and therapy resistance. Similarly, S1184 association with CHEK2 (S379) and BRAF (S729) links it to MAPK-driven proliferation and DDR, potentially exacerbating genomic instability in breast cancer (Sect. 3.5). The co-regulation of S363 and S1184 with ERBB3 and EPHA2 further implicates receptor tyrosine kinase signaling (Sect. 3.8). These pathways converge on tumor progression by enhancing proliferation, survival, and resistance to endocrine therapies like tamoxifen, particularly in luminal A subtypes.

In summary, phosphorylation at S363 likely disrupts ARID1A SWI/SNF interactions, impairing chromatin remodeling and promoting oncogenesis, while S696 and S1184 enhance DDR and MAPK signaling, driving genomic instability and therapy resistance. The phosphorylation status of S363 and S1184 in breast cancer underscores their role as molecular switches in pathogenesis, offering potential biomarkers and therapeutic targets.

## Limitations of the study

While this study provides a novel phosphosite-centric framework to explore the regulatory network of ARID1A, several limitations must be acknowledged that may influence the interpretation and generalization of our findings. First, our reliance on heterogeneous public phosphoproteomic datasets—spanning diverse experimental platforms, conditions, and cell types—introduces variability despite stringent quality controls (Class-1 phosphosites: localization probability ≥ 75%, A-score ≥ 13) and multi-layered filtering. Complete normalization across such datasets is challenging without access to raw data and uniform reprocessing pipelines. Second, our correlation-based approach does not confirm causation, and the functional roles of many correlated phosphosites (PsOPs) remain speculative without biochemical validation, especially for poorly characterized PsOPs. Additionally, known ARID1A regulatory sites (e.g., S363, S1184, and S696) were underrepresented, likely due to mass spectrometry limitations in detecting low-abundance or transient phosphorylation events. Finally, while we identified key biological processes linked to correlated phosphosites, their causal impact on pathways like DNA damage response requires experimental confirmation. In summary, although our study highlights potential regulatory roles for uncharacterized ARID1A phosphosites (e.g., S363, S1184, and S696), targeted studies involving time-resolved phosphoproteomics, phosphosite mutagenesis, and functional assays are essential to validate these findings and elucidate ARID1A’s phosphorylation network.

## Conclusions and future directions

This analysis unveils the intricate co-differentially regulated protein network of ARID1A, illuminating its potential role in chromatin remodeling and DNA damage response (DDR) within breast cancer. Through systematic phosphoproteomic analysis, we identified three predominant ARID1A phosphosites—S363, S1184, and S696. MAPK14, CDK16, and MAPK9 were predicted as putative upstream kinases and the binary interactors and phosphatases among the co-differentially regulated proteins were elucidated. Association of ARID1A with DDR proteins, including TP53BP1, TOP2A, CHEK2, and NBN, highlights its integration into the double-strand break (DSB) repair network. Dysregulation of these phosphorylation events and their downstream pathways drives cancer progression, therapeutic resistance, and poor prognosis.

Notably, ARID1A phosphorylation exhibits tumor-specific patterns, with S363 and S1184 showing marked upregulation in breast cancer. This hyperphosphorylation suggests a shift in ARID1A regulatory function, potentially enhancing oncogenic signaling or impairing its tumor-suppressive activity. These site-specific modifications align with the observed interplay between ARID1A and MAPK-driven pathways. The co-regulation of ARID1A with SWI/SNF and DDR proteins further positions it as a molecular hub, linking chromatin dynamics to genome integrity—a balance frequently disrupted in breast cancer. These findings have significant therapeutic implications. The upregulation of S363 and S1184, alongside ARID1A interactions with BRD9 and other SWI/SNF components, suggests that targeting ARID1A phosphorylation or its associated pathways potentially through MAPK inhibitors could restore its tumor-suppressive function in breast cancer. Such strategies could be particularly effective in patients with hyperphosphorylated ARID1A, offering a precision medicine approach to improve outcomes in endocrine-resistant cases.

In summary, this study advances our understanding of phosphorylation-dependent regulation of ARID1A, providing critical insights into its contributions to cancer biology and disease progression in breast cancer. Future research should prioritize experimental validation of these kinase-substrate interactions, using techniques such as kinase assays or phosphomimetic mutants, to elucidate their effects on transcriptional regulation and chromatin structure. Additionally, developing targeted therapies to modulate these pathways could unlock novel treatment avenues, addressing the unmet needs of patients with ARID1A-dysregulated cancers.

## Supplementary Information

Supplementary Material 1.

## Data Availability

Data is provided within the manuscript or supplementary information files.
